# The Mark of the Beast

**Published:** 1891-02-21

**Authors:** 


### The Mark of the Beast.

SOME NOTES ON CRIMINAL ANTHROPOLOGY.

"The child, what will he become?" asks a popular poster, and goes on to depict the child's development to the two possible extremes of honoured age and disreputable beggary. The notion is effective, and contains a fair amount of truth, but, as a matter of fact, even the child's countenance, if one could examine it closely enough, would show whether it was likely to take the upward or the downward path. Ruskin says, "All that a man is (as with a crow or a duckling) was in the shell of him, mental vulgarity and all." (One might add to this "moral depravity and all.") "The moral question is what of the good in him you can get at and nourish, what of the bad chain down." This is the utmost that can be done; natures cannot be changed, but they may be modified for good or evil, as sickly children, who would die if neglected, may be with care reared to maturity, and to a usefulness of their own. It will not be the usefulness of the healthy and energetic, but it will serve some purpose. Only, it is folly to pretend that the strong and the weak, the good and the evil, are fundamentally the same.

It is by this assumption that we have for generations blundered in our treatment of criminals. Taking it for granted that it is as easy for the child to advance to "honoured age" as to "disreputable beggary," we have boxed its ears and shut it up in the dark because it took the wrong turning. Some discipline is necessary in all cases, but in enforcing it let us not forget that the child's destiny was partly settled before it was born. It is an ascertained fact that the children of elderly parents are often weak in intellect; nay, it is believed that a fit of intoxication or some severe mental strain at the mysterious moment when life begins, may mark a child for ever with some weakness of mind or morals. The history of the famous "Jukes" family, of America, is a striking instance of criminal heredity. The founder of the family, a descendant of the early Dutch settlers, was born between 1720 and 1740. He was a hardy backwoodsman, but given to drink, and averse to steady toil. The descendants of this man have been traced with more or less completeness through five generations. The history of 700 is known, and though the family has not lacked honest members, it has been on the whole, as a contemporary writer says, "a family of criminals and prostitutes, of vagabonds and paupers." Of the men of the family only twenty were skilled workmen, and ten of these had learned their trade in prison. Idleness and vice seem to be ineradicable in the tribe.

Close and experienced observers learn in time to know prisoners by head-mark, and to trace the connection between physical and moral degeneracy. Lombroso expresses the opinion that "in general born criminals have projecting ears, thick hair, a thin beard, projecting frontal eminences, enormous jaws, a square and projecting chin, large cheek-bones, and frequent gesticulation. It is, in short, a type resembling the Mongolian, or sometimes the Negroid." This description is, perhaps, too definite, but there certainly seems to be a family likeness among criminals. As Hepworth Dixon points out, though the population of Milbank is continually changing, a stranger visiting the place year after year might think it was unchanged. The individual goes, the type remains unaltered as new-comers arrive to fill the vacant place.

More unvarying than the physical, is the moral type, which in its developments seems to touch childishness on the one hand and lunacy on the other. The vanity of criminals is of a markedly insane type. A murderer, who killed three rich men, had previously been overheard to say, "I want to do something great; oh, I shall be talked about." A Russian lad of nineteen horrified all St. Petersburg by the murder of a whole family. When he was told of the sensation his crime had created, his comment was: "Now my school-fellows will see how unfair it was of them to say that I should never be heard of." Such a way of looking at crime shows more than wickedness; it betrays a moral incapacity, a *lacuna* in the whole nature. It is egotism pushed to its most deadly extreme. It must, however, be remembered that in criminal society the greater the crime the greater the distinction. The man who is imprisoned for theft will boast to his fellow-criminals that he is a murderer in the same spirit of social vanity that makes a militia captain claim to be "in the army," or induces an impecunious lady to hire diamonds to go to Court in. All three wish to make an impression on their acquaintances.

Akin to this is the restlessness of temperament that causes mutinies in prison, these unreasoning outbreaks of temper, sullenness, or open rebellion, which are so difficult to explain. Sometimes they are barometric; it has been noticed that outbreaks of insubordination are commonest in spring and autumn, as if the equinox had something to do with it. Sometimes it seems to be constitutional on the part of the prisoner, who can often predict that he will "break out" that day or the next, but apparently cannot help it. Dostoieffsky, the Russian novelist, some years of whose life were spent in Siberian prisons, says on the subject: "This unexpected explosion is the anguished convulsive manifestation of personality, an instinctive melancholy, a desire to affirm the degraded *ego*, an emotion which obscures the judgment." Perhaps it is not necessary to analyse the matter thus finely. Are not these outbreaks simply a protest against the monotony of prison life, the lawlessness of the undisciplined nature that has never yielded obedience, crying out against an existence of which obedience and order are the essential parts? This lack of discipline is of the essence of criminal nature; it shows itself in good as well as in evil, and when accompanied by repentance comes out in the most impracticable sentimentality. A lady of our acquaintance took a repentant thief as a servant. The woman proved honest, loyal, and devoted, yet she was all but impossible as a member of any orderly household. Her mistress fell ill; she had a sister and a nurse to attend to her, and the servant was supposed to be in the kitchen making beef-tea. Not at all; she insisted on kneeling outside the bedroom door praying aloud for Mrs. E's. life. Such conduct was touching, but it was more likely to do harm than good, and one cannot think it was wholly free from vanity. She wanted people to know how devoted she was.

Perhaps the most extreme case of this sentimentality on record is that of a German, who, having murdered his sweetheart, went back to her house, at some danger to himself, to liberate her canary, which might otherwise have starved in its cage. "Lacenaire," we are told by Mr. Havelock Ellis, "on the same day that he committed a murder, risked his own life to save that of a cat." By what measure shall one judge natures so strangely compounded? They are not cruel in the ordinary sense, only egoistic in the highest degree. If you offend them, if even you stand between them and anything they covet, it seems to them the most simple, reasonable, natural thing in the world to remove you. It is not a question of malice in the ordinary sense of the term, only a matter of convenience, and to the cat or the canary, who do not happen to be inconvenient at the moment the murderer can show even a maudlin tenderness. Fundamentally, the cause may be said to be lack of balanced imagination. The brutality of the criminal lunatic who, having killed a family of children, amused himself by tossing their corpses in the air; the irritability of him who killed his companion "because he snored so loud;" the cynicism of him who called out to a friend in the crowd, "Hallo! do you know I've just been condemned to death!" are of the same web as the examples of emotion we have quoted. All alike show lack of imagination, of that sense of proportion in human affairs, of that comprehension of others' rights, and sympathy with others' feelings, which keeps ordinary men from wrong. At his best, as at his worst, the criminal is self-centred, an egotist; and only in so far as he can be taught that he is a member of society at large, and owes a duty to it, is his reformation possible.

